# Metabolic Effects of Metformin in the Failing Heart

**DOI:** 10.3390/ijms19102869

**Published:** 2018-09-21

**Authors:** Aleksandra Dziubak, Grażyna Wójcicka, Andrzej Wojtak, Jerzy Bełtowski

**Affiliations:** 1 Department of Pathophysiology, Medical University of Lublin, ul. Jaczewskiego 8b, 20-090 Lublin, Poland; a_dziubak@interia.pl (A.D.); grazyna.wojcicka@umlub.pl (G.W.); 2 Department of Vascular Surgery, Medical University of Lubin, 20-090 Lublin, Poland; andrzejwojtak@umlub.pl

**Keywords:** Metformin, diabetes, heart failure, glucotoxicity, lipotoxicity

## Abstract

Accumulating evidence shows that metformin is an insulin-sensitizing antidiabetic drug widely used in the treatment of type 2 diabetes mellitus (T2DM), which can exert favorable effects on cardiovascular risk and may be safely used in patients with heart failure (HF), and even able to reduce the incidence of HF and to reduce HF mortality. In failing hearts, metformin improves myocardial energy metabolic status through the activation of AMP (adenosine monophosphate)-activated protein kinase (AMPK) and the regulation of lipid and glucose metabolism. By increasing nitric oxide (NO) bioavailability, limiting interstitial fibrosis, reducing the deposition of advanced glycation end-products (AGEs), and inhibiting myocardial cell apoptosis metformin reduces cardiac remodeling and hypertrophy, and thereby preserves left ventricular systolic and diastolic functions. While a lot of preclinical and clinical studies showed the cardiovascular safety of metformin therapy in diabetic patients and HF, to confirm observed benefits, the specific large-scale trials configured for HF development in diabetic patients as a primary endpoints are necessary.

## 1. Introduction

As first reported in the Framingham study, type 2 diabetes mellitus (T2DM) is not only an independent risk factor for cardiovascular diseases (CVD), but is also associated with a higher incidence of heart failure (HF). The risk of heart failure in patients with diabetes is almost twice higher for men and five times for women when compared with the general population. Moreover, patients with diabetes account for one third of all cases of HF [1]. As the prevalence of diabetes rapidly rises worldwide, the incidence of HF consequently increases in parallel [2]. The concomitance of diabetes and heart failure significantly worsens a prognosis in this group of patients. There is a tight positive correlation between hyperglycemia and heart failure development and progression. Diabetic patients have an 8% increase in the risk of developing HF with every 1% elevation of glycated hemoglobin (HbA1c) [3,4]. While the development of HF is related to poor glycemic control, even patients with good metabolic profiles have an increased risk [5]. Nowadays, there are many classes of anti-diabetic medication; nevertheless their role in the therapy of diabetes with heart failure is still undefined. Some drugs used for diabetes have been associated with increased rate of hospitalization for HF, despite its neutral or positive effect on overall risk of major cardiovascular events. For example, saxagliptin and alogliptin should be avoided in patients with diabetes and HF and pioglitazone is contraindicated in this population [6,7]. On the other hand, it is suggested that some antihyperglycemic drugs can reduce the rate of hospitalization due to heart failure and reduce mortality. These benefits have been proven for empagliflozin, an inhibitor of the sodium-glucose co-transporter 2 (SGLT-2) [8] and metformin, the biguanide derivative.

Metformin (1,1-dimethylbiguanide), an insulin-sensitizer, is currently a basis drug used in the treatment of T2DM recommended in all stages of therapy, in monotherapy and in combination with other oral antihyperglycemic drugs and insulin [9,10,11]. The biguanide derivatives have been implemented to diabetes mellitus treatment in the 1950’s [12]. Phenformin and buformin have been withdrawn two decades later due to frequent cases of life-threatening lactic acidosis [13,14,15]. Metformin, as a safer, less lipophilic derivative, after 20 years of its use in Europe was registered in USA in 1995 [16], after publishing the results of De Fronzo and Goodman trial, that validated the safety and benefits of this drug [17].

The largest randomized, multicenter clinical study, The United Kingdom Prospective Diabetes Study, was the first to demonstrate the ability of metformin to reduce the risk of macroangiopathy in patients with newly diagnosed diabetes and obesity or overweight, more than sulfonylureas or insulin. In comparison with conventional diet therapy, metformin reduced the risk of any diabetes-related complication by 32%, the risk of death due to diabetes by 42% and the risk of myocardial infarction by 39%. The risk of hypoglycemia was lower for intensive metformin treatment than for intensive treatment with sulfonyloureas or insulin [18].

It should be emphasized that, according to the current Summary of Product Characteristics, metformin is contraindicated in heart failure [10]. However, the results presented in the latest reports verify the contraindication of metformin in this setting. The recent trials demonstrated that metformin does not enhance the risk of lactic acidosis, and can be safety used in patients with type 2 diabetes mellitus and early heart failure [19]. This review presents the molecular basis of antihyperglycemic mechanism of action of metformin, its impact on myocardial metabolism and cardioprotective mechanisms that are independent of glycemic control. While the cardio-protective properties of metformin are well established, the role of metformin therapy in patients with diabetes and concomitant HF is still the subject of much controversy. In this review, we highlight the metabolic effects of this anti-diabetic agent in failing heart that could explain the benefit and safety of metformin therapy in this population.

## 2. Mechanisms of Antihyperglycemic Action

Metformin effectively reduces fasting and postprandial glucose and diminishes HbA1c level by more than 1% [20]. In cells the drug acts mainly by transient inhibition of oxidative phosphorylation at the level of respiratory-chain complex I [21]. In vitro studies provide evidence that there is a slow permeation of the drug across the inner mitochondrial membrane where it directly inhibits complex 1 in a time-dependent, self-limiting manner. The positive charge of metformin accounts for its accumulation within the mitochondrial matrix. As the matrix concentration of the drug increases, progressive inhibition of the respiratory chain leads to a drop in membrane potential, which prevents further accumulation of the drug [22]. As a result, an electron transport in the inner mitochondrial membrane is inhibited. Consequently, the synthesis of ATP (adenosine triphosphate) decreases and the level of AMP (adenosine monophosphate) increases in the cell [14,21]. AMP activates the AMP-activated protein kinase (AMPK), the main enzyme that regulates energy balance in the cell and makes it able to adapt to energy deficiency conditions [14,23]. AMPK is composed of three subunits: Catalytic α-subunit and two regulatory subunits: β and γ. AMP activates AMPK by binding with γ subunit, that changes α subunit conformation making it more susceptible to phosphorylation by LKB1 (liver kinase B1). The other enzyme able to activate AMPK is CaMKKβ (calcium/calmodulin dependent protein kinase kinase β). CaMKKβ activates α subunit in conditions of increased Ca^2+^ concentration inside the cell [24].

Physiologically, the activation of AMPK induced by processes that decrease ATP levels in the cell, such as physical effort, hypoglycemia, ischemia, and hypoxia. AMPK activity can also be stimulated by hormones that regulate energy balance, like ghrelin, leptin (in skeletal muscle), and adiponectin [25,26]. Additionally, AMPK agonists include also AICAR (5-aminoimidazole-4-carboxamide ribonucleotide), resveratrol, and thiazolidinediones. By contrast, resistin and endocannabinoids inhibit enzyme activity [27].

**Figure 1:**
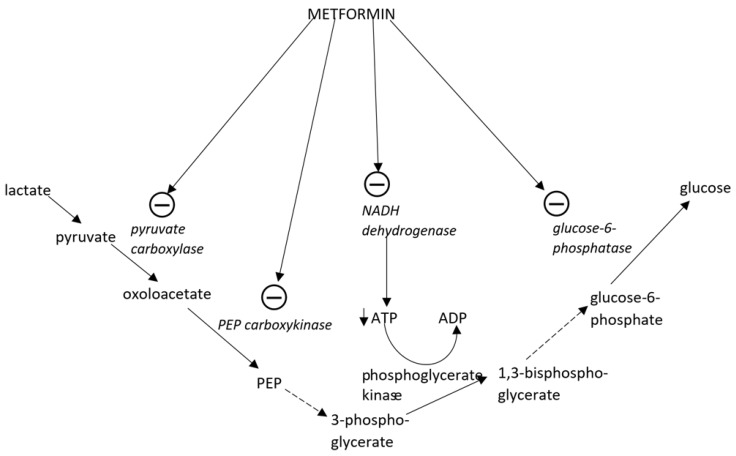
The impact of metformin on gluconeogenesis. ADP, adenosine diphosphate; ATP, adenosine triphosphate; NADH, nicotinamide adenine dinucleotide phosphate; PEP, phosphoenolpyruvate. Circles with “−” inside indicate inhibitory effect on the enzyme. Continuous and dotted arrows represent one- and multiple-step reactions, respectively.

Metformin-induced energy deficiency in the cell causes AMPK activation and inhibits the processes in which ATP is consumed such as gluconeogenesis, cholesterol, and fatty acids synthesis or glycogen synthesis (Figure 1). Simultaneously activated metabolic pathways producing ATP like free fatty acids (FFA) oxidation in the liver and skeletal muscles or glycolysis [12,23].

In the liver inhibition of gluconeogenesis by metformin is caused not only by the reduced availability of ATP [11], but also by blocking lactate uptake into hepatocytes and the inhibition of the main enzymes of gluconeogenesis such as (1) pyruvate carboxylase (PC), that converts pyruvate into oxaloacetate; (2) phosphoenolpyruvate carboxykinase (PEPCK), that converts oxaloacetate into PEP (phosphoenolpyruvate); (3) glucose-6-phosphatase, that hydrolyses glucose-6-phosphate to glucose [28,29,30]. Through the activation of insulin receptor substrate two (IRS-2), metformin enhances GLUT-1 (glucose transporter 1)-mediated glucose transport into hepatocytes [31].

In the liver, metformin also affects lipid synthesis and catabolism. By activating AMPK, metformin down-regulates *SREBP-1* (sterol regulatory element binding protein 1) gene expression. *SREBP-1* regulates transcription of genes encoding lipogenic enzymes, such as fatty acid synthase (FAS). In addition, AMPK activation inhibits acetyl-CoA carboxylase (ACC) and thereby malonyl-CoA synthesis. Malonyl-CoA is the substrate for fatty acid synthesis and an inhibitor of CPT-1 (carnitine palmitoyltransferase 1) [32], the enzyme that transports fatty acids to mitochondria [33]. Therefore, suppression of malonyl-CoA synthesis decreases production of fatty acids and triglycerides in hepatocytes, increasing at the same time FFA oxidation in mitochondria. Moreover, AMPK activation decreases cholesterol synthesis in hepatocytes as a result of HMG-CoA reductase (3-hydroxy-3-methyl-glutaryl-coenzyme A reductase) inhibition [34].

In the skeletal muscle cells, metformin increases insulin-dependent glucose uptake and its utilization in anaerobic glycolysis [35,36]. Furthermore, metformin enhances anaerobic glycolysis in other tissues [11,37].

In adipocytes metformin regulates adipogenesis, lipolysis, and FFA oxidation, thus reduces the release of FFA from the adipose tissue. The reduction of plasma FFA level is associated with more efficient glucose uptake by the cells of peripheral tissues [38,39].

In the intestines metformin inhibits absorption of glucose and other simple carbohydrates [40]. In addition, it has been proved that metformin is able to improve the action of intestine-pancreas axis (incretin effect). The drug is considered to directly increase secretion of glucagon-like peptide-1 (GLP-1) from enterocytes. In addition through inhibiting dipeptidyl peptidase-4 (DPP-4), an enzyme responsible for incretin degradation, metformin may prolong the duration and activity of endogenous incretins [37,41]. Metformin may also exert its glucose-lowering and insulin-sensitizing action via modifying microbiota composition [42]. Furthermore, it may contribute to the improvement of pancreatic β-cell function by reducing gluco and lipotoxicity [43,44].

Metformin is slowly absorbed from the proximal small intestine and the rate of absorption depends on the dose. The drug accumulates in the gastrointestinal tissues that may contribute to the gastrointestinal-associated side effects [45]. Intestinal absorption of metformin is probably facilitated by plasma membrane monoamine transporter (PMAT) as well as OCT1 and OCT3 (organic cation transporters) [46]. OCTs play important role in distribution of metformin over body tissues, such as intestine, kidney, and liver [46,47]. Steady-state plasma concentrations of metformin may vary from one to 10 μM [45]. The drug is not metabolized in the liver and is excreted unchanged in urine. It is eliminated by active tubular secretion, with a half-life of approximately five h [46,48]. The uptake of the drug from circulation into renal epithelial cells is mediated through OCT2 and its excretion from the tubular cell into the urine is facilitated by MATE1 and MATE2-K (multidrug and toxin extrusion proteins) [46]. It is suggested that genetic polymorphisms in drug transporters are associated with wide variations in pharmacokinetics profile and a large inter-individual variability in metformin response [47,48].

## 3. Diabetic Cardiomyopathy

Diabetes mellitus is an independent risk factor of congestive heart failure [49]. Coronary artery disease, arterial hypertension, and diabetic cardiomyopathy are the main factors that contribute to the development of HF in diabetes. The risk of heart failure in T2DM remains higher compared to non-DM (diabetes mellitus) individuals after adjustment for other risk factors such as serum cholesterol levels, hypertension, or coronary artery disease [50,51]. On the other hand, the presence of DM worsens prognosis in the heart failure population in which the mortality rates are significantly higher than in the non-DM population [52].

Diabetic cardiomyopathy (DMC) is a damage of myocardium that occurs in diabetes independently of concomitant disorders like hypertension and ischemic heart disease [53,54]. Cardiomyopathy is characterized by increased myocardial stiffness and left ventricular mass (cardiac hypertrophy) [55,56]. At the beginning, it is an asymptomatic impairment of left ventricular diastolic function that leads to symptomatic diastolic dysfunction and finally the systolic function becomes impaired too [57,58,59]. Diastolic left ventricular (LV) dysfunction (a restrictive phenotype) has been linked to concentric hypertrophy of LV, and eccentric remodeling of LV has been linked to systolic dysfunction (a dilated phenotype). When becoming symptomatic, diabetic patients with a restrictive phenotype of DMC present heart failure with preserved ejection fraction (HFpEF), which is typically characterized by small LV cavity, normal LV ejection fraction (EF), thick LV walls, elevated LV filling pressure, and large left atrium. Whereas, in patients with a dilated cardiomyopathy, clinical heart failure with reduced ejection fraction (HFrEF) is recognized. These patients have abnormal-sized LV and abnormal LV ejection fraction [60].

On pathological examination in DMC, there was myocardial hypertrophy, fibrosis and microvascular wall thickening. At the tissue level, DMC is characterized by interstitial or replacement fibrosis, increases in extracellular matrix [60,61], and deposition of advanced glycation end-products (AGEs). All these changes contribute to reduced diastolic and systolic compliance and ventricular hypertrophy. At the cellular level in DM-related cardiomyopathy, the hypertrophy as well as fragmentation and degeneration of myocytes are observed [59,60]. The latter is particularly strongly expressed in the dilated type of DMC [60]. At the molecular level, DMC is connected with the disturbance of calcium transport into cardiomyocytes, disorders of fatty acid metabolism and decrease in Na^+^, K^+^-ATPase activity [59,62].

The underlying pathophysiology of DMC is multifactorial but can largely be attributed to metabolic derangements that accompany T2DM like insulin resistance, hyperinsulinemia, hyperglycemia and dyslipidemia. These perturbations disturb myocardial metabolism and impair endothelial function of coronary micro vessels (microangiopathy). The main feature of endothelial dysfunction is reduced bioavailability of NO (nitric oxide), which leads to disrupted nitric oxide (NO)-cyclic guanosine monophosphate (cGMP)-protein kinase G (PKG) signaling pathway and predisposes to concentric LV remodeling and diastolic stiffness of LV (restrictive phenotype). Capillary rarefaction, defined as a decrease in the number of perfused capillaries in an area of tissue, leads to hypoxia and oxidative stress, cardiomyocyte death, and DMC with dilated phenotype [60].

Vascular endothelial dysfunction, manifested with impaired NO production, is thought to be one of key risk factors for diabetic cardiomyopathy. Nitric oxide plays a key role in maintaining cardiac and vascular homeostasis, which is achieved when there is a balance between the generation of NO and superoxide anion (O_2_^−^). The cardiac endothelium comprises the endothelial cells of the coronary microvasculature, of the endocardium and the intramyocardial capillaries. The heart cardiomyocytes lies in close proximity to endothelial cells (maximum 3 μm from endothelial cells). This specific location allows adequate blood supply and facilitates the bidirectional communication among those cells [63]. In the heart NO is a powerful anti-hyperthrophic and anti-fibrotic agent. These protective effects of NO are mediated predominantly via activation of intracellular sGC (soluble guanylate cyclase) and subsequent cGMP (cyclic guanosine monophosphate) generation [64]. The reduction of NO-dependent signaling from endothelium to cardiomyocytes might lead to the impairment of NO-sGC signaling pathway and then to cardiac remodeling and fibrosis—consequently to ventricle stiffness, impaired relaxation, and cardiac dysfunction [65,66]. NO exerts its anti-fibrotic effects by the inhibition of the transforming growth factor (TGF-β1)-induced cardiac fibroblast proliferation, transformation and collagen synthesis, and trough activation of cGMP/PKG pathway [67].

Impaired insulin metabolic signaling in the heart results in metabolic stress which manifests in disturbed substrate uptake and utilization, disturbed energy status, mitochondrial dysfunction, increased gene expression of the stiffer protein, and increased fibroblast activity leading to collagen deposition in an extracellular matrix that contributes to myocardial remodeling and diastolic dysfunction [51,68,69,70]. Insulin resistance-associated hiperinsulinemia can stimulate cardiomyocyte hypertrophy by binding to the IGF-1 (insulin growth factor 1) receptor, as well as stimulating a PI-3K/Akt (phosphatidylinositol-3-kinase/Akt) signaling pathway [51,71].

Most studies discussing diabetic cardiomyopathy have focused only on left ventricle, but the effects on the right ventricle in diabetic cardiomyopathy have not been studied extensively. Nevertheless, from a pathophysiological point of view, all proposed mechanisms leading to LV impairment in type 2 diabetes are systemic changes and therefore might also hamper right ventricular (RV) structure and function. Indeed, impairment of RV function has been reported in Zucker diabetic fatty rats [72] and in human studies [73]. Moreover, impaired parameters of RV diastolic function were shown in patients with type 2 diabetes suggesting that the similar mechanisms responsible for LV stiffness also affect the right ventricle. Alternatively, RV involvement could be the consequence of left ventricle changes, e.g., diffuse fibrotic processes that take place in diabetes could affect the function of both ventricles. Another possibility is that in diabetes, microangiopathy of the lung capillary [74] may cause increased right ventricle afterload leading to its dysfunction.

## 4. Metabolic Disorders in Diabetic Cardiomyopathy

Metabolic disorders that are observed in cardiomyocytes in patients with diabetes arise from toxic action of both high levels of FFA (lipotoxicity) and hyperglycemia (glucotoxicity). Physiologically, at normal work loads in aerobic conditions, the energy is acquired by cardiomyocytes mainly from fatty acid β-oxidation (60–90%). Only a small percentage of energy is produced in glycolysis and pyruvate oxidation [68,75,76,77]. Both β-oxidation and glycolysis leads to the synthesis of acetyl-CoA, which is next oxidized in citric acid cycle [77]. Glycolysis requires less oxygen to synthesis one mole of ATP than FFA oxidation, hence glucose is used as a basic source of energy during ischemia and oxygen deficiency [76,78]. Metabolic disorders that occur in diabetes significantly impair the ability of the heart to adapt to overload conditions, e.g., physical activity [79,80].

### 4.1. Lipotoxicity 

**Figure 2:**
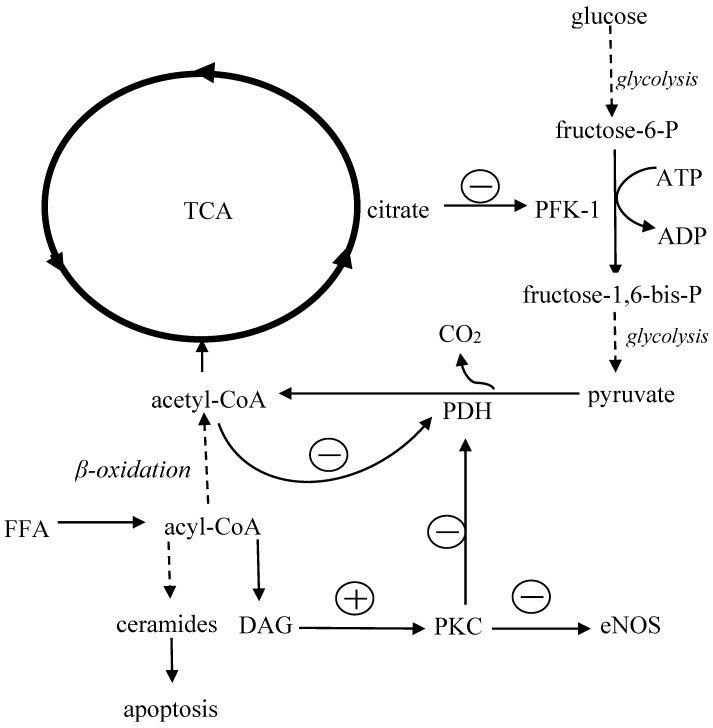
The impact of FFA overload on metabolism of the heart. Acetyl-CoA, acetyl coenzyme A; acyl-CoA, acyl coenzyme A; ADP, adenosine diphosphate; ATP, adenosine triphosphate; DAG, diacylglycerol; eNOS, endothelial nitric oxide synthase; FFA, free fatty acids; PDH, pyruvate dehydrogenase; PFK-1, phosphofructokinase 1; PKC, protein kinase C; and TCA, tricarboxylic acid cycle. Continuous and dotted arrows represent one- and multiple-step reactions, respectively. Arrows with circles containing “+” or “−” represent stimulatory and inhibitory effects, respectively.

The impact of FFA overload on metabolism of the heart shows in Figure 2. In general, the main mechanism leading to insulin resistance is the excessive accumulation of adipose tissue, especially visceral adipose tissue, which is less sensitive to insulin but more susceptible to catecholamines; both these abnormalities result in accelerated lipolysis. Increased release of non-esterified fatty acids from adipocytes raises the level of circulating FFA [81,82] and impairs insulin signaling in the liver, skeletal muscles and adipose tissue [83,84,85,86]. It results in a decreased uptake and consumption of glucose in muscles, abolished the inhibitory effect of insulin on glucose synthesis in hepatocytes [87,88] and facilitated the uptake and accumulation of FFA into cardiomyocytes [49]. Increased activity of lipogenesis-regulating enzymes such as glycerol phosphate acyltransferase (GPAT) causes myocardial triglyceride accumulation, heart steatosis, and eventually cell death [89]. Excess of fatty acids inhibits cardiac glucose utilization. This is because the long-term exposure to high FFA level increases acetyl-CoA concentration in mitochondria, which inhibits the activity of glycolytic enzymes such as phosphofructokinase 1 (PFK-1) and pyruvate dehydrogenase (PDH). As a consequence, glucose oxidation decreases, which results in the accumulation of glycolysis intermediate products such as glucose-6-phosphate, fructose-6phosphate, pyruvate, and lactate [90].

When FFA uptake exceeds the ability of their utilization in the heart, FFA accumulates in the cytoplasm. Within the intracellular compartment, fatty acids (long chain fatty acyl-CoA) are converted into toxic ceramides, which induce cell apoptosis, or into diacylglycerol (DAG), which activates protein kinase C (PKC) [59,90]. PKC inactivates pyruvate dehydrogenase in cardiomyocytes and thus impairs glucose oxidation [91,92]. Furthermore, in endothelium PKC reduces the activity of eNOS (endothelial nitric oxide synthase), increases synthesis of endothelin 1 as well as stimulates NADPH oxidase (nicotinamide adenine dinucleotide phosphate oxidase), which generates reactive oxygen species (ROS), further inactivating nitric oxide [92,93,94,95]. High FFA levels may also affect structure and function of cardiomyocyte membranes and raise intracellular calcium concentration leading to disturbances of conduction and myocardial contractility [96,97,98].

### 4.2. Glucotoxicity

Glucose accumulation in cardiomyocytes inhibits FFA oxidation by increasing intracellular malonyl-CoA. Malonyl-CoA is an inhibitor of carnitine palmitoyltransferase I, that is responsible for transport of FFA into mitochondria [90,99]. In addition, hyperglycemia blocks FFA oxidation at the level of gene expression via inhibiting PPARα (peroxisome proliferator-activated receptor alpha) that regulates the expression of enzymes necessary for mitochondrial β-oxidation. In addition, intensified glycation of proteins involved in the insulin signaling pathways such as IRS that reduces cell sensitivity to insulin [90]. Insulin resistance is associated with reduced expression of glucose transporters *GLUT-1* and *GLUT-4* [83,100].

Moreover, advanced glycation end products generated during hyperglycemia induces the synthesis of reactive oxygen species that impair the function of ion transporters and mitochondria, affect calcium transport between cell compartments, and initiate apoptosis [90]. Moreover, glycation of collagen increases myocardial stiffness and impairs diastolic function [59]. Changes in extracellular matrix disturb conduction of impulses thus results in arrhythmias [61,101]. Oxidative stress caused by hyperglycemia and AGEs contributes to damage of autonomic neurons that regulate contractility of heart and coronary vessels. Parasympathetic neurons are damaged at first, resulting in the predominance of a sympathetic nervous system. Then sympathetic neurons are damaged too. Autonomic neuropathy leads to other disorders such as resting tachycardia and asymptomatic ischemia of myocardium [102].

## 5. Cardioprotective Effect of Metformin

### 5.1. Glucose and FFA Metabolism

**Figure 3:**
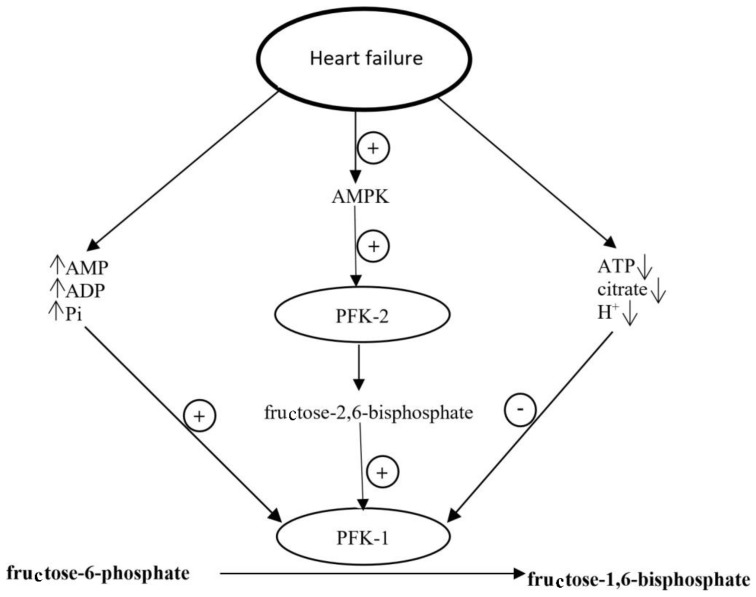
Regulation of PFK activity in heart failure. ADP, adenosine diphosphate; AMP, adenosine monophosphate; AMPK, AMP activated protein kinase; ATP, adenosine triphosphate; PFK-1, phosphofructokinase 1; PFK-2, phosphofructokinase 2; and Pi, inorganic phosphate. Circles with “+” and “−” represent stimulatory and inhibitory effects, respectively. ↑ and ↓ represent increase and decrease in the concentration of specific compound, respectively.

In failing heart, the activation of metabolic pathways controlled by AMPK in cardiomyocytes is a mechanism that allows adapting to conditions under which the energy generation is reduced [103,104]. In this setting, changes in the level of factors regulating phosphofructokinase 1 activity have been observed. For example, the level of AMP, ADP (adenosine diphosphate) and inorganic phosphates, those are PFK-1 allosteric activators, increases and the level of PFK-1 inhibitors, such as ATP, citrate, and H^+^ ions decreases [103]. Moreover, AMPK activates phosphofructokinase 2 (PFK-2), leading to the increased concentration of fructose 2,6-biphosphate, another PFK-1 activator produced in cardiomyocytes (Figure 3). As PFK-1 is the rate-limiting enzyme of glycolysis, its activation causes intensification of glycolysis [26,78,103]. Furthermore, AMPK activation leads to the translocation of *GLUT-4* to cardiomyocyte membrane and accelerates insulin-dependent glucose uptake [90,103]. In vitro studies confirm that increase in PFK-1 activator level and decreased level of citrate, phosphocreatine, and H^+^ ions are accompanied by twofold higher glucose utilization than in control cardiomyocytes, in spite of comparable ATP generation and oxygen utilization [103].

In vitro study showed that metformin added to the incubation medium significantly increased glucose uptake by stimulating the phosphoinositide 3-kinase (PI 3-K)-protein kinase B/Akt pathway and AMPK activation. This positive effect was observed both in insulin resistant cardiomyocytes and in cells with normal insulin sensitivity [105]. Thus, in diabetic hearts due to AMPK activation, the correct glucose metabolism can be restored.

If in diabetic hearts the activation of the AMP-activated kinase cascade increases energy production through intensification of glucose oxidation, paradoxically it could lead to inhibition of β-oxidation and accumulation of FFA in cardiomyocytes and cardiac steatosis. Indeed, it has been proved that obese patients with impaired glucose tolerance or type 2 diabetes can have even twofold higher triglycerides level in the heart than healthy people, even without symptoms of left ventricular dysfunction [50]. Hence a question arises, will metformin treatment not worsen lipotoxicity resulting from accumulation of fatty acids and triglycerides? No unequivocal answer has been found so far. Nevertheless, in vitro studies showed that small doses of metformin did not worsen, but even protected heart muscle from fat-induced apoptosis. It has been observed that AMPK activated acyl-CoA oxidation and inhibited SPT (serine palmitoyltransferase), which is responsible for ceramide synthesis, as well as inhibited caspase 3, is involved in myocytes apoptosis [93].

**Figure 4:**
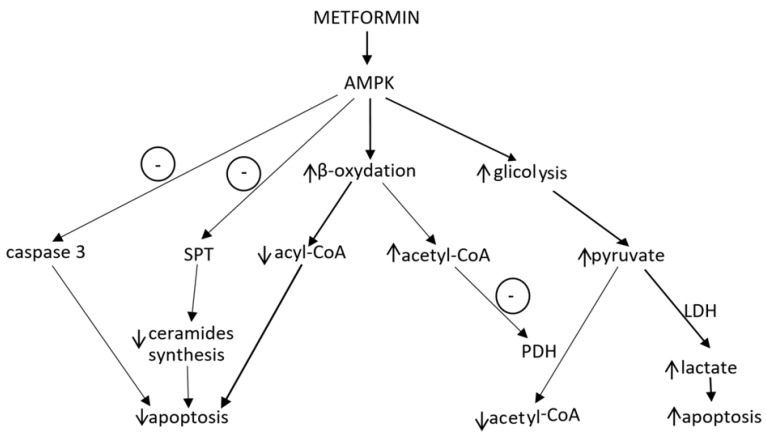
The effect of metformin on apoptosis. Acetyl-CoA, acetyl coenzyme A; acyl-CoA, acyl coenzyme A; AMPK, AMP-activated protein kinase; LDH, lactate dehydrogenase; PDH, pyruvate dehydrogenase; and SPT, serine palmitoyltransferase. ↑ and ↓ represent increase and decrease in the concentration of specific compound, respectively. Circles with “−” represent inhibitory effect.

On the other hand, there are studies showing that metformin at high doses may increase the number of cells undergoing apoptosis as a result of FFA accumulation [106]. As already mentioned, metformin-induced AMPK phosphorylation enhances not only glucose transport and glycolysis, but also fatty acid uptake and β-oxidation (Figure 4). Furthermore, acetyl-CoA synthetized during β-oxidation blocks the stage of glycolysis in which pyruvate is oxidized by PDH. This inhibition results in a sudden release of lactate dehydrogenase (LDH), converting pyruvate into lactate. Accumulation of lactate causes pH reduction, Ca^2+^ overload, and cell death. This effect is not observed when glucose is removed from the incubation medium. Lactate synthesis and pH reduction are also observed when lower doses of metformin are used, but metformin at low concentrations has no influence on cardiomyocytes survival [106].

In addition, in vivo studies showed that chronic administration of metformin declined FFA level in the plasma and increased their oxidation in non-diabetic cardiomyocytes. At the same time, the inhibition of glucose oxidation was observed, probably as a result of the glycolysis-blocking effect of metformin, directly or indirectly, by acetyl-CoA synthesized during FFA oxidation. Increased β-oxidation and inhibition of glycolysis had no influence on heart failure progression [107].

### 5.2. Protein Synthesis

In vitro studies confirmed that pharmacological activation of AMPK could prevent heart muscle hypertrophy by inhibiting protein synthesis [108,109]. It has been postulated that there are two signaling pathways that play key roles in protein synthesis: eEF-2 (eukaryotic elongation factor 2) pathway and p70S6 (ribosomal protein S6) kinase pathway [109]. eEF-2 regulates the movement of the ribosome along the mRNA during elongation [110], while p70S6 kinase phosphorylases eEF-2 kinase [111] and ribosomal protein S6 [112]. It has been proven that metformin-induced AMPK activation inhibits protein synthesis by increasing the level of phosphorylated, (inactive) eEF-2 protein and decreasing phosphorylation of p70S6 kinase, leading to activation of the eEF-2 kinase, as well as to the inhibition of ribosomal protein S6 activity [108].

### 5.3. Mitochondrial Function

The other mechanism explaining the beneficial effect of metformin on metabolism of failing heart is improvement of mitochondria function in cardiomyocytes. In experimental heart failure induced by myocardial ischemia in mice, four-week administration of small doses of metformin significantly improved left ventricular function and structure and increased animal survival almost by 47%. This effect was related to elevated AMPK phosphorylation and increased the expression of *eNOS* and *PGC-1α* (peroxisome proliferator-activated receptor gamma coactivator 1-alpha) [113]. Both *eNOS* and *PGC-1α* are important regulators of biogenesis and function of mitochondria [114,115,116], and their activation improves oxidative metabolism in cardiomyocytes, i.e., increases ATP synthesis and restores normal ratio of ATP synthesis/oxygen use. By contrast, in other studies performed in transgenic animals with inactive *AMPKα2* and *eNOS* genes, this cardioprotective effect of metformin was not observed [113].

### 5.4. iNOS Activity and Calcium Ions Transport

In the heart tissue, there are expressed three isoforms of NO-synthase (NOS)—endothelial NOS (eNOS), neuronal NOS (nNOS), and inducible NOS (iNOS). Endothelial NOS is principally expressed in endothelial cells and cardiomyocytes and is a key source of NO [64]. Neuronal NOS is mainly located in cardiomyocytes. Expression of iNOS has been found in immune cells, cardiomyocytes, and activated fibroblast. In heart muscle, the main physiological role is played by NO generated by eNOS and nNOS [117].

Increased expression of inducible NO synthase (iNOS) is observed during myocardial ischemia in both damaged and non-damaged areas in cardiomyocytes, endothelial cells, and macrophages. It is suggested that the enzyme may be involved in the development of late complications of myocardial infarction such as congestive heart failure [118]. It has been proven that excessive synthesis of NO by iNOS contributes to the impairment of diastolic and systolic LV function. Selective iNOS inhibitors, such as SMTU (S-methylisothiourea) and aminoguanidine improve myocardial contractility. On the other hand, L-arginine, an unselective iNOS substrate, causes the opposite effect, disturbing myocardial function [119]. In contrast to eNOS that synthesizes small nanomolar amounts of NO, iNOS produces NO in micromolar concentration [120]. High cellular NO level, via cGMP, a second messenger, activates PKG and cGMP-activated phosphodiesterase. PKG blocks L-type calcium channels and consequently decreases calcium influx into the cells, while phosphodiesterase breaks down cAMP [119]. Decreases in the intracellular Ca^2+^ and cAMP concentrations may contribute to impaired myocardial contractility under conditions of iNOS-related NO overproduction [49,119].

Long term exposition of intra and extracellular structures to toxic NO concentrations can cause tyrosine nitration in specific proteins and induce apoptosis [119]. Metformin in the in vitro studies has been shown to inhibit lipopolysaccharide-induced elevation of iNOS mRNA (messenger ribonucleic acid) expression in macrophages. Therefore, metformin via blocking the excessive NO generation can reduce formation of cardiotoxic peroxynitrite. This effect was partially dependent on AMPK phosphorylation [121]. It should be emphasized that iNOS activity is regulated at the level of gene expression, mainly by proinflammatory cytokines, such as IL-1β (interleukin-1β) [122]. In vitro studies proved that, through AMPK activation, metformin inhibited synthesis of IL-1β in activated macrophages [121]. It is also noteworthy, that metformin, by activating AMPK, facilitates eNOS phosphorylation/activity [123] and reduces the activity of TGF-β1, which is the basic pro-fibrotic growth factor in the cardiovascular system myocardium [124].

### 5.5. Collagen Synthesis and Glycation

In diabetes, the important mechanism leading to impaired heart muscle relaxation is the interstitial accumulation of collagen and its non-enzymatic glycation [125]. Metformin may inhibit both of them.

The preclinical study conducted in normoglycemic mice with left ventricular pressure overload showed that metformin can inhibit collagen synthesis in myocardium, which contributes to left ventricular dimension reduction and significantly decreases diastolic pressure in the LV. This effect resulted from the ability of metformin to inhibit the synthesis of TGF-β1 in myocardium and was independent of its glucose and insulin-lowering effects [126]. The same authors, in another study, showed that metformin in cultured fibroblasts may inhibit phosphorylation of Smad3 factor and its translocation to the nucleus stimulated by TGF-β1 [126]. The TGF-β1/Smad3 signal pathway displays a significant role in the regulation of the expression of extracellular matrix protein genes [127]. Thus, its inhibition by metformin explains the reduction of collagen synthesis. This effect of metformin was not related to AMPK activation [126].

**Figure 5:**
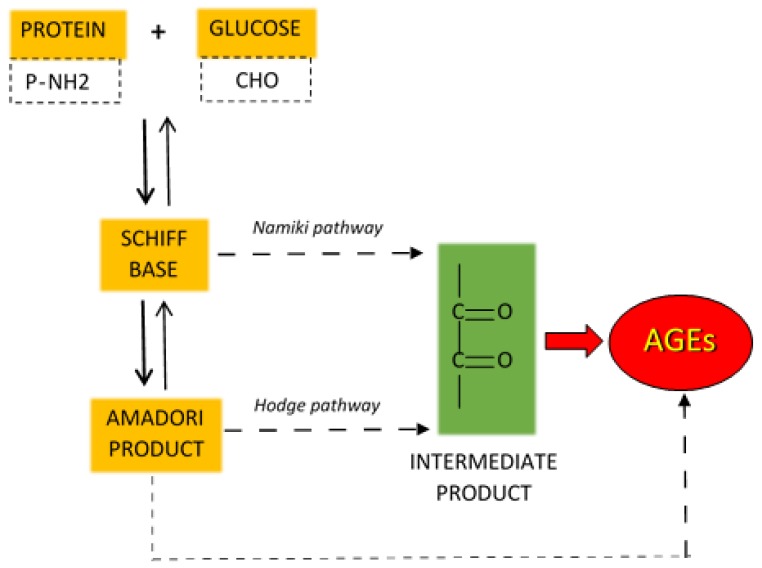
Maillard reaction. Carbonyl groups of glucose react with amino groups of proteins (both in dotted frames) to form Schiff bases (yellow) which then are spontaneously converted to Amadori products (yellow). Both of them contribute to formation of low molecular weight reactive dicarbonyls (intermediate products, green) which bind to proteins forming advacned glycation end products (AGEs, red). Continuous and dotted arrows represent one- and multiple-step reactions, respectively.

**Figure 6:**
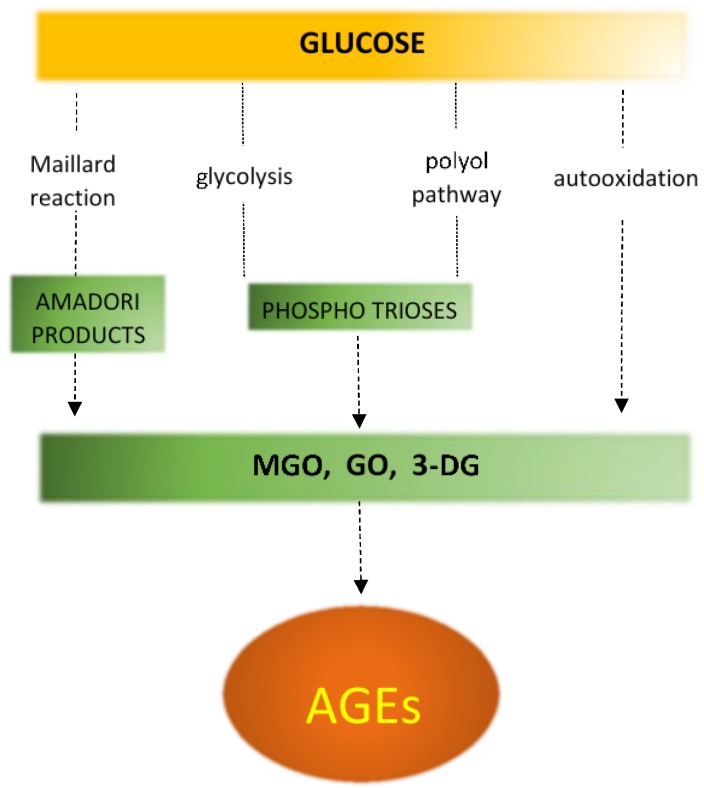
Different mechanisms of AGE formation. MGO—methylglyoxal, GO—glyoxal, 3-DG—3-deoxyglucosome. Initial, intermediary and terminal steps are shown in yellow, green and orange, respectively. Dotted arrows and lines represent multiple-step reactions.

Non-enzymatic protein glycation (NEG) plays an important role in the development of structural and functional abnormalities in diabetic heart. In this process, in the Maillard reaction, carbonyl groups of reducing sugars attach to free amino groups of amino acids (lysine and arginine) of proteins as well as of nucleic acids or phospholipids (Figure 5) [128,129,130]. In diabetes, in addition to sustained hyperglycemia, inflammation, oxidative stress and polyol pathway are also the important processes that can exacerbate protein glycation (Figure 6) [131,132]. The Maillard reaction leads to synthesis of reactive dicarbonyls, such as glyoxal, methylglyoxal, and 3-deoxyglucosone [129,130]. Subsequently these compounds may covalently bind to amino groups of proteins forming advanced glycation end products [129]. AGEs may alter protein structure by producing crosslinks within or between proteins. AGEs do not disassociate and accumulate permanently in long-living protein such as collagen in myocardium and vessels, increasing their stiffness and impairing relaxation [59,130].

Glycation of collagen increases its resistance to the enzymatic breakdown, leading to collagen accumulation and fibrosis [131,133,134]. Metformin is able to inhibit generation of AGEs by the mechanism related to direct neutralization of reactive dicarbonyls, through binding the guanidine group of drug to α-dicarbonyl group of methylglyoxal. Furthermore, metformin activates the glyoxolase, an enzyme that converts methylglyoxal to D-lactate. This beneficial effect was confirmed in numerous preclinical studies [135,136]. For example, it was documented that metformin is able to protect apolipoprotein A-I of HDL (high density lipoprotein) fraction from glycation induced by methylglyoxal [137]. Metformin also inhibits modification of apolipoprotein B in LDL (low density lipoprotein) fraction by glycolaldehyde or methylglyoxal [138], and inhibits AGEs generation in macrophages during incubation with glyoxal [139]. In the rats with streptozotocin-induced diabetes, as a result of administration of metformin, smaller amounts of AGEs in the kidney cortex, lens and sciatic nerve were observed [140]. Moreover, lower levels of circulating AGEs were found in diabetic patients treated with metformin [141]. In addition, a study performed on diabetic dogs revealed that metformin could also prevent glycation of collagen in the heart muscle and thereby could reduce heart stiffness. It has been demonstrated that metformin can restore diastolic myocardial function in these animals, reflected by the normalization of end-diastolic pressure and end-diastolic volume. In the heart of animals given metformin, there was observed reduced AGEs binding to collagen fibers. It is interesting that although the total amount of collagen in the heart of these animals remained unchanged, metformin significantly improved heart function [125].

### 5.6. Apoptosis

Protective effect of metformin on heart muscle is also mediated by the inhibition of cardiomyocyte apoptosis. It has been demonstrated that metformin, through AMPK activation, prevents apoptosis of cardiomyocytes during their incubation with H_2_O_2_ [104]. Similarly, an in vivo study showed that metformin reduced the number of dead cardiomyocytes in the hearts of dogs with experimental heart failure. After four weeks treatment with metformin, improvement of LV function and hemodynamic parameters was observed. These cardioprotective, antiapoptotic properties were partially dependent on AMPK activation and *eNOS* expression, phosphorylation/activation, resulting in the increase in NO synthesis [104].

The evidence of cardioprotective effect of metformin was also found in a study on rats with heart damage caused by subcutaneous injection of isoproterenol [142]. Isoproterenol induces changes consistent with those that appear in patients during myocardial infarction, like cardiomyocytes necrosis, cardiac arrhythmias, decrease in arterial pressure indices, including LV contractility and relaxation, and increase in the left ventricular end-diastolic pressure [143,144]. These changes can lead to dysfunction of the heart, mainly of the left ventricle. Metformin declined heart weight, increased cardiomyocyte survival, and reduced intercellular matrix accumulation, as well as decelerated heart rate in rats subjected to isoproterenol-induced myocardial toxicity. Additionally, the average blood pressure, left ventricular end-diastolic pressure and left ventricular systolic pressure were normalized [142].

## 6. Metformin in Heart Failure

The experimental studies and clinical observations supply a growing number of arguments that confirm safety and benefits of metformin in patients with heart failure. For example, in study involving patients with heart failure and type 2 diabetes, at the first stage of antihyperglycemic treatment, metformin, in monotherapy or in combination with sulfonylureas, reduced the total mortality and risk of death or hospitalization compared to sulfonylureas therapy alone [145]. This result has been confirmed in other trials that involved patients with newly diagnosed type 2 diabetes and heart failure. Metformin, both in monotherapy and polytherapy reduced mortality in comparison with traditional treatment based only on diet and life style changes. The effect of metformin was independent of glycemic control and the BMI (body mass index) value, and has not been observed after thiazolidinediones or insulin administration [146]. In another non-randomized study, during two years observation, treatment with metformin reduced mortality of ambulatory patients with diabetes and heart failure [147]. There is also evidence that metformin monotherapy is associated with a lower risk of heart failure development in patients with recent diabetes than sulfonylurea monotherapy, even at high doses [148]. In addition, it has been proven that metformin use in monotherapy or in combination with other antihyperglycemic drugs such as sulfonylurea derivatives, thiazolidinediones, or insulin have a positive effect on patients with advanced heart failure in III and IV New York Heart Association (NYHA) class. After consideration of differences between groups, the results showed a tendency to reduction of all-cause mortality, reduction of composed endpoint (death or urgent heart transplant) risk and to increase in left ventricular ejection fraction after metformin therapy in comparison to other oral antihyperglycemic drugs or insulin [149].

According to the recommendations of the international guidelines, metformin should not be used in patients with diabetes and heart failure because of a risk of lactic acidosis. However, the connection between metformin blood level and lactate blood level during lactic acidosis is not observed in clinical practice. Lactic acidosis is rather the result of comorbid diseases and the risk of lactic acidosis is similar whether metformin is used or not [150,151]. Metanalysis of nine observational studies demonstrated that metformin can be safely used in heart failure. In none of the trials metformin increased mortality of patients with decreased left ventricular ejection fraction, even in those with III and IV NYHA class of heart failure and patients with chronic kidney failure. The analysis also showed that metformin reduced the risk of all-cause hospitalization and the risk of hospitalization due to heart failure. There was no trial to demonstrate that metformin treatment was associated with a higher risk of lactic acidosis than other antihyperglycemic drugs [151].

In conclusion, based on experimental and clinical data, metformin therapy in diabetes and concomitant heart failure is not associated with higher risk and the benefits of its usage exceed the potential danger. Moreover, it is increasingly suggested that metformin should be a first choice of treatment in this group of patients [152,153]. Therefore, the revising of contraindications of metformin use seems to be reasonable.

As the cardioprotective properties of metformin are not associated with an antihyperglycemic effect, the drug could be also beneficial for patients with heart failure without concomitant diabetes. 

## Abbreviations

ACC-1	acetyl-CoA carboxylase 1
ACC-2	acetyl-CoA carboxylase 2
ADP	adenosine diphosphate
AICAR	5-aminoimidazole-4-carboxamide ribonucleotide
AGEs	advanced glycation end products
AMP	adenosine monophosphate
AMPK	AMP-activated protein kinase
ATP	adenosine triphosphate
BMI	body mass index
CaMKKβ	calcium/calmodulin dependent protein kinase kinase β
cAMP	cyclic adenosine monophosphate
cGMP	cyclic guanosine monophosphate
CPT-1	carnitine palmitoyltransferase 1
CVD	cardiovascular diseases
DAG	diacylglycerol
DMC	diabetic cardiomyopathy
DPP-4	dipeptidyl peptidase-4
eEF-2	eukaryotic elongation factor 2
EF	ejection fraction
eNOS	endothelial nitric oxide synthase
FAS	fatty acid synthase
FFA	free fatty acids
GLP-1	glucagon-like peptide-1
*GLUT-1*	glucose transporter 1
*GLUT-4*	glucose transporter 4
GPAT	glycerol phosphate acyltransferase
HbA1c	glycosylated hemoglobin
HDL	high density lipoprotein
HF	heart failure
HFpEF	heart failure with preserved ejection fraction
HFrEF	heart failure with reduced ejection fraction
HMG-CoA	3-hydroxy-3-methyl-glutaryl-coenzyme A
IGF-1	insulin growth factor 1
IL-1β	interleukin 1β
IRS-2	insulin receptor substrate 2
iNOS	inducible NO synthase
LDH	lactate dehydrogenase
LDL	low density lipoprotein
LKB1	liver kinase B1
LV	left ventricular
MATE1	multidrug and toxin extrusion protein 1
MATE2-K	multidrug and toxin extrusion protein 2-K
mRNA	messenger ribonucleic acid
NEG	non-enzymatic protein glycation
nNOS	neuronal NO synthase
NO	nitric oxide
NYHA	New York Heart Association
OCT 1	organic cation transporter 1
OCT 2	organic cation transporter 2
OCT 3	organic cation transporter 3
p70S6	ribosomal protein S6
PC	pyruvate carboxylase
PDE	cGMP-regulated phosphodiesterase
PDH	pyruvate dehydrogenase
PEP	phosphoenolpyruvate
PEPCK	phosphoenolpyruvate carboxykinase
PFK-1	phosphofructokinase 1
PFK-2	phosphofructokinase 2
*PGC-1α*	peroxisome proliferator-activated receptor gamma coactivator 1-alpha
PKC	protein kinase C
PKG	cGMP-dependent protein kinase
PI-3K	phosphatidylinositol-3-kinases
PMAT	plasma membrane monoamine transporter
*PPARα*	peroxisome proliferator-activated receptor alpha
RV	right ventricular
SERCA2a	sarco/endoplasmic reticulum Ca2^+^-ATPase
sGC	soluble guanylate cyclase
SMTU	S-methylisothiourea
SPT	serine palmitoyltransferase
*SREBP-1*	sterol regulatory element binding protein 1
T2DM	type 2 diabetes mellitus
TGF-β1	transforming growth factor β1

## References

[1] Kinsara A.J., Ismail Y.M. (2018). Metformin in heart failure patients. Indian Heart J..

[2] Lambadiari V., Dimitriadis G., Kadoglou N.P.E. (2018). The impact of oral anti-diabetic medications on heart failure: Lessons learned from preclinical studies. Heart Fail. Rev..

[3] Iribarren C., Karter A.J., Go A.S., Ferrara A., Liu J.Y., Sidney S., Selby J.V. (2001). Glycemic control and heart failure among adult patients with diabetes. Circulation.

[4] Palazzuoli A., Ceccarelli E., Ruocco G., Nuti R. (2018). Clinical impact of oral antidiabetic medications in heart failure patients. Heart Fail. Rev..

[5] Schaan B.D., de Figueiredo Neto J.A., Moreira L.B., Ledur P., Mattos L.A.P., Magnoni D., Precoma D.B., Machado C.A., da Silva Brasileiro A.L., Pena F.M. (2017). Diabetes and cardiovascular events in high-risk patients: Insights from a multicenter registry in a middle-income country. Diabetes Res. Clin. Pract..

[6] Eleftheriadou I., Grigoropoulou P., Liberopoulos E., Liatis S., Kokkinos A., Tentolouris N. (2018). Update on cardiovascular effects of older and newer anti-diabetic medications. Curr. Med. Chem..

[7] LeBras M.H., Barry A.R., Koshman S.L. (2017). Cardiovascular safety outcomes of new antidiabetic therapies. Am. J. Health Syst. Pharm..

[8] Fitchett D., Zinman B., Wanner C., Lachin J.M., Hantel S., Salsali A., Johansen O.E., Woerle H.J., Broedl U.C., Inzucchi S.E. (2016). EMPA-REG OUTCOME^®^ trial investigators. Heart failure outcomes with empagliflozin in patients with type 2 diabetes at high cardiovascular risk: Results of the EMPA-REG OUTCOME^®^ trial. Eur. Heart J..

[9] Bajaj S. (2015). RSSDI clinical practice recommendations for the management of type 2 diabetes mellitus. Int. J. Diabetes Dev. Ctries.

[10] Rojas L.B.A., Gomes M.B. (2013). Metformin: An old but still the best treatment for type 2 diabetes. Diabetol. Metab. Syndr..

[11] Zheng J., Woo S.L., Hu X., Botchlett R., Chen L., Huo Y., Wu C. (2015). Metformin and metabolic diseases: A focus on hepatic aspects. Front. Med..

[12] Dowling R., Goodwin P.J., Stambolic V. (2011). Understanding the benefit of metformin use in cancer treatment. BMC Med..

[13] Anisimov V.N. (2014). Do metformin a real anticarcinogen? A critical reappraisal of experimental data. Ann. Transl. Med..

[14] Pryor R., Cabreiro F. (2015). Repurposing metformin: An old drug with new tricks in its binding pockets. Biochem. J..

[15] White J.R. (2014). A brief history of the development of diabetes medications. Diabetes Spectr..

[16] Witters L.A. (2001). The blooming of the French lilac. J. Clin. Investig..

[17] DeFronzo R.A., Goodman A.M. (1995). The Multicenter Metformin Study Group. Efficacy of metformin in patients with non-insulin-dependent diabetes mellitus. N. Engl. J. Med..

[18] UK Prospective Diabetes Study (UKPDS) Group (1998). Effect of intensive blood-glucose control with metformin on complications in overweight patients with type 2 diabetes (UKPDS 34). Lancet.

[19] Kappel B.A., Marx N., Federici M. (2015). Oral hypoglycemic agents and the heart failure conundrum: Lessons from and for outcome trials. Nutr. Metab. Cardiovasc. Dis..

[20] Vilar L., Canadas V., Arruda M.J., Arahata C., Agra R., Pontes L., Montenegro L., Vilar C.F., Silva L.M., Albuquerque J.L. (2010). Comparison of metformin, gliclazide MR and rosiglitazone in monotherapy and in combination for type 2 diabetes. Arq. Bras. Endocrinol. Metabol..

[21] Viollet B., Guigas B., Sanz Garcia N., Leclerc J., Foretz M., Andreelli F. (2012). Cellular and molecular mechanisms of metformin: An overview. Clin. Sci..

[22] Owen M.R., Doran E., Halestrap A.P. (2000). Evidence that metformin exerts its anti-diabetic effects through inhibition of complex 1 of the mitochondrial respiratory chain. Biochem. J..

[23] Towler M.C., Hardie D.G. (2007). AMP-activated protein kinase in metabolic control and insulin signaling. Circ. Res..

[24] Zou M.H., Wu Y. (2008). AMP-activated protein kinase activation as a strategy for protecting vascular endothelial function. Clin. Exp. Pharmacol. Physiol..

[25] Hardie D.G. (2014). AMP-activated protein kinase: Maintaining energy homeostasis at the cellular and whole body levels. Annu. Rev. Nutr..

[26] Kim T.T., Dyck J.R. (2015). Is AMPK the savior of the failing heart?. Trends Endocrinol. Metab..

[27] Viollet B., Guigas B., Leclerc J., Hébrard S., Lantier L., Mounier R., Andreelli F., Foretz M. (2009). AMP-activated protein kinase in the regulation of hepatic energy metabolism: From physiology to therapeutic perspectives. Acta Physiol..

[28] Im I., Jang M., Park S.J., Lee S.H., Choi J.H., Yoo H.W., Kim S., Han Y.M. (2015). Mitochondrial respiratory defect causes dysfunctional lactate turnover via AMP-activated protein kinase activation in human-induced pluripotent stem cell-derived hepatocytes. J. Biol. Chem..

[29] Steinberg G.R., Kemp B.E. (2009). AMPK in health and disease. Physiol. Rev..

[30] Chung S.T., Chacko S.K., Sunehag A.L., Haymond M.W. (2015). Measurements of gluconeogenesis and glycogenolysis: A methodological review. Diabetes.

[31] Gunton J.E., Delhanty P.J., Takahashi S., Baxter R.C. (2003). Metformin rapidly increases insulin receptor activation in human liver and signals preferentially through insulin-receptor substrate-2. J. Clin. Endocrinol. Metab..

[32] Zhou G., Myers R., Li Y., Chen Y., Shen X., Fenyk-Melody J., Wu M., Ventre J., Doebber T., Fujii N. (2001). Role of AMP-activated protein kinase in mechanism of metformin action. J. Clin. Investig..

[33] Zang Y., Wang T., Xie W., Wang-Fischer Y.L., Getty L., Han J., Corkey B.E., Guo W. (2005). Regulation of acetyl CoA carboxylase and carnitine palmitoyl transferase-1 in rat adipocytes. Obes. Res..

[34] Viollet B., Foretz M., Guigas B., Horman S., Dentin R., Bertrand L., Hue L., Andreelli F. (2006). Activation of AMP-activated protein kinase in the liver: A new strategy for the management of metabolic hepatic disorders. J. Physiol..

[35] Kristensen J.M., Treebak J.T., Schjerling P., Goodyear L., Wojtaszewski J.F. (2014). Two weeks of metformin treatment induces AMPK-dependent enhancement of insulin-stimulated glucose uptake in mouse soleus muscle. Am. J. Physiol. Endocrinol. Metab..

[36] Protti A., Properzi P., Magnoni S., Santini A., Langer T., Guenzani S., Ferrero S., Bassani G., Stocchetti N., Gattinoni L. (2016). Skeletal muscle lactate overproduction during metformin intoxication: An animal study with reverse microdialysis. Toxicol. Lett..

[37] McCreight L.J., Bailey C.J., Pearson E.R. (2016). Metformin and the gastrointestinal tract. Diabetologia.

[38] Moreno-Navarrete J.M., Ortega F.J., Rodríguez-Hermosa J.I., Sabater M., Pardo G., Ricart W., Fernández-Real J.M. (2011). OCT1 expression in adipocytes could contribute to increased metformin action in obese subjects. Diabetes.

[39] Abbasi F., Carantoni M., Chen Y.-D.I., Reaven G.M. (1998). Further evidence for a central role of adipose tissue in the antihyperglycemic effect of metformin. Diabetes Care.

[40] Gu S., Shi J., Tang Z., Sawhney M., Hu H., Shi L., Fonseca V., Dong H. (2015). Comparison of glucose lowering effect of metformin and acarbose in type 2 diabetes mellitus: A. meta-analysis. PLoS ONE.

[41] Mannucci E., Ognibene A., Cremasco F., Bardini G., Mencucci A., Pierazzuoli E., Ciani S., Messeri G., Rotella C.M. (2001). Effect of metformin on glucagon-like peptide 1 (GLP-1) and leptin levels in obese nondiabetic subjects. Diabetes Care.

[42] Zhou Z.Y., Ren L.W., Zhan P., Yang H.Y., Chai D.D., Yu Z.W. (2016). Metformin exerts glucose-lowering action in high-fat fed mice via attenuating endotoxemia and enhancing insulin signaling. Acta Pharmacol. Sin..

[43] Lastra G., Manrique C.M., Hayden M.R. (2006). The role of beta-cell dysfunction in the cardiometabolic syndrome. J. Cardiometab. Syndr..

[44] Lupi R., Del Guerra S., Fierabracci V., Marselli L., Novelli M., Patanè G., Boggi U., Mosca F., Piro S., Del Prato S. (2002). Lipotoxicity in human pancreatic islets and the protective effect of metformin. Diabetes.

[45] Kinaan M., Ding H., Triggle C.R. (2015). Metformin: An old drug for the treatment of diabetes but a new drug for the protection of the endothelium. Med. Princ. Pract..

[46] Gong L., Goswamic S., Giacominic K.M., Altmana R.B., Klein T.E. (2012). Metformin pathways: Pharmacokinetics and pharmacodynamics. Pharmacogenet. Genom..

[47] Yoon H., Cho H.Y., Yoo H.D., Kim S.M., Lee Y.B. (2013). Influences of organic cation transporter polymorphisms on the population pharmacokinetics of metformin in healthy subjects. AAPS J..

[48] Goswami S., Yee S.W., Stocker S., Mosley J.D., Kubo M., Castro R., Mefford J.A., Wen C., Liang X., Witte J. (2014). Genetic variants in transcription factors are associated with the pharmacokinetics and pharmacodynamics of metformin. Clin. Pharmacol. Ther..

[49] Falcão-Pires I., Leite-Moreira A.F. (2012). Diabetic cardiomyopathy: Understanding the molecular and cellular basis to progress in diagnosis and treatment. Heart Fail. Rev..

[50] McGavock J.M., Lingvay I., Zib I., Tillery T., Salas N., Unger R., Levine B.D., Raskin P., Victor R.G., Szczepaniak L.S. (2007). Cardiac steatosis in diabetes mellitus: A 1H-magnetic resonance spectroscopy study. Circulation.

[51] Riehle C., Abel E.D. (2016). Insulin signaling and heart failure. Circ. Res..

[52] Asleh R., Sheikh-Ahmad M., Briasoulis A., Kushwaha S.S. (2018). The influence of anti-hyperglycemic drug therapy on cardiovascular and heart failure outcomes in patients with type 2 diabetes mellitus. Heart Fail. Rev..

[53] Zlobine I., Gopal K., Ussher J.R. (2016). Lipotoxicity in obesity and diabetes-related cardiac dysfunction. Biochim. Biophys. Acta.

[54] Kandula V., Kosuru R., Li H., Yan D., Zhu Q., Lian Q., Ge R.S., Xia Z., Irwin M.G. (2016). Forkhead box transcription factor 1: Role in the pathogenesis of diabetic cardiomyopathy. Cardiovasc. Diabetol..

[55] Carugo S., Giannattasio C., Calchera I., Paleari F., Gorgoglione M.G., Grappiolo A., Gamba P., Rovaris G., Failla M., Mancia G. (2001). Progression of functional and structural cardiac alterations in young normotensive uncomplicated patients with type 1 diabetes mellitus. J. Hypertens..

[56] Joffe I.I., Travers K.E., Perreault-Micale C.L., Hampton T., Katz S.E., Morgan J.P., Douglas P.S. (1999). Abnormal cardiac function in the streptozotocin-induced non-insulin-dependent diabetic rat: Noninvasive assessment with doppler echocardiography and contribution of the nitric oxide pathway. J. Am. Coll. Cardiol..

[57] Mesquita E.T., Jorge A.J. (2013). Understanding asymptomatic diastolic dysfunction in clinical practice. Arq. Bras. Cardiol..

[58] Boudina S., Abel E.D. (2007). Diabetic cardiomyopathy revisited. Circulation.

[59] Dei Cas A., Spigoni V., Ridolfi V., Metra M. (2013). Diabetes and chronic heart failure: From diabetic cardiomyopathy to therapeutic approach. Endocr. Metab. Immune Disord. Drug Targets.

[60] Seferović P.M., Paulus W.J. (2015). Clinical diabetic cardiomyopathy: A two-faced disease with restrictive and dilated phenotypes. Eur. Heart J..

[61] Wong T.C., Piehler K.M., Kang I.A., Kadakkal A., Kellman P., Schwartzman D.S., Mulukutla S.R., Simon M.A., Shroff S.G., Kuller L.H. (2014). Myocardial extracellular volume fraction quantified by cardiovascular magnetic resonance is increased in diabetes and associated with mortality and incident heart failure admission. Eur. Heart J..

[62] Liu C.C., Fry N.A., Hamilton E.J., Chia K.K., Garcia A., Karimi Galougahi K., Figtree G.A., Clarke R.J., Bundgaard H., Rasmussen H.H. (2013). Redox-dependent regulation of the Na^+^-K^+^; pump: New twists to an old target for treatment of heart failure. J. Mol. Cell. Cardiol..

[63] Tschöpe C., Van Linthout S. (2014). New insights in (inter)cellular mechanisms by heart failure with preserved ejection fraction. Curr. Heart Fail. Rep..

[64] Ritchie R.H., Drummond G.R., Sobey C.G., De Silva T.M., Kemp-Harper B.K. (2017). The opposing roles of NO and oxidative stress in cardiovascular disease. Pharmacol. Res..

[65] Paulus W.J., Tschöpe C. (2013). A novel paradigm for heart failure with preserved ejection fraction: Comorbidities drive myocardial dysfunction and remodeling through coronary microvascular endothelial inflammation. J. Am. Coll. Cardiol..

[66] Zuo L., Chuang C.C., Hemmelgarn B.T., Best T.M. (2015). Heart failure with preserved ejection fraction: Defining the function of ROS and NO. J. Appl. Physiol..

[67] Chen J., Shearer G.C., Chen Q., Healy C.L., Beyer A.J., Nareddy V.B., Gerdes A.M., Harris W.S., O’Connell T.D., Wang D. (2011). Omega-3 fatty acids prevent pressure overload-induced cardiac fibrosis through activation of cyclic GMP/protein kinase G signaling in cardiac fibroblasts. Circulation.

[68] Hunter W.G., Kelly J.P., McGarrah R.W., Kraus W.E., Shah S.H. (2016). Metabolic dysfunction in heart failure: Diagnostic, prognostic, and pathophysiologic insights from metabolomic profiling. Curr. Heart Fail. Rep..

[69] Mizushige K., Yao L., Noma T., Kiyomoto H., Yu Y., Hosomi N., Ohmori K., Matsuo H. (2000). Alteration in left ventricular diastolic filling and accumulation of myocardial collagen at insulin-resistant prediabetic stage of a type II diabetic rat model. Circulation.

[70] Shiomi T., Tsutsui H., Ikeuchi M., Matsusaka H., Hayashidani S., Suematsu N., Wen J., Kubota T., Takeshita A. (2003). Streptozotocin-induced hyperglycemia exacerbates left ventricular remodeling and failure after experimental myocardial infarction. J. Am. Coll. Cardiol..

[71] O’Neill B.T., Abel E.D. (2005). Akt1 in the cardiovascular system: Friend or foe?. J. Clin. Investig..

[72] Van den Brom C.E., Bosmans J.W., Vlasblom R., Handoko L.M., Huisman M.C., Lubberink M., Molthoff C.F., Lammertsma A.A., Ouwens M.D., Diamant M. (2010). Diabetic cardiomyopathy in Zucker diabetic fatty rats: The forgotten right ventricle. Cardiovasc. Diabetol..

[73] Widya R.L., van der Meer R.W., Smit J.W., Rijzewijk L.J., Diamant M., Bax J.J., de Roos A., Lamb H.J. (2013). Right ventricular involvement in diabetic cardiomyopathy. Diabetes Care.

[74] Klein O.L., Krishnan J.A., Glick S., Smith L.J. (2010). Systematic review of the association between lung function and Type 2 diabetes mellitus. Diabet. Med..

[75] Bayeva M., Sawicki K.T., Ardehali H. (2013). Taking diabetes to heart—deregulation of myocardial lipid metabolism in diabetic cardiomyopathy. J. Am. Heart Assoc..

[76] Lionetti V., Stanley W.C., Recchia F.A. (2011). Modulating fatty acid oxidation in heart failure. Cardiovasc. Res..

[77] Sankaralingam S., Lopaschuk G.D. (2015). Cardiac energy metabolic alterations in pressure overload–induced left and right heart failure. Pulm. Circ..

[78] Ingwall J.S. (2009). Energy metabolism in heart failure and remodeling. Cardiovasc. Res..

[79] Kolwicz S.C., Purohit S., Tian R. (2013). Cardiac metabolism and its interactions with contraction, growth, and survival of the cardiomyocte. Circ. Res..

[80] Gibb A.A., Hill B.G. (2018). Metabolic coordination of physiological and pathological cardiac remodeling. Circ. Res..

[81] Preis S.R., Massaro J.M., Robins S.J., Hoffmann U., Vasan R.S., Irlbeck T., Meigs J.B., Sutherland P., D’Agostino R.B., O’Donnell C.J. (2010). Abdominal subcutaneous and visceral adipose tissue and insulin resistance in the Framingham Heart Study. Obesity.

[82] Wajchenberg B.L. (2000). Subcutaneous and visceral adipose tissue: Their relation to the metabolic syndrome. Endocr. Rev..

[83] Petersen K.F., Shulman G.I. (2006). Etiology of insulin resistance. Am. J. Med..

[84] Aguirre G.A., Rodríguez De Ita J., de la Garza R.G., Castilla-Cortazar I. (2016). Insulin-like growth factor-1 deficiency and metabolic syndrome. J. Transl. Med..

[85] Nguyen M.T., Satoh H., Favelyukis S., Babendure J.L., Imamura T., Sbodio J.I., Zalevsky J., Dahiyat B.I., Chi N.W., Olefsky J.M. (2005). JNK and tumor necrosis factor-alpha mediate free fatty acid-induced insulin resistance in 3T3-L1 adipocytes. J. Biol. Chem..

[86] Pansuria M., Xi H., Li L., Yang X.F., Wang H. (2012). Insulin resistance, metabolic stress, and atherosclerosis. Front. Biosci. (Schol. Ed.).

[87] Czech M.P., Tencerova M., Pedersen D.J., Aouadi M. (2013). Insulin signalling mechanisms for triacylglycerol storage. Diabetologia.

[88] Foster M.T., Pagliassotti M.J. (2012). Metabolic alterations following visceral fat removal and expansion. Beyond anatomic location. Adipocyte.

[89] Zhou Y.T., Grayburn P., Karim A., Shimabukuro M., Higa M., Baetens D., Orci L., Unger R.H. (2000). Lipotoxic heart disease in obese rats: Implications for human obesity. Proc. Natl. Acad. Sci. USA.

[90] Young M.E., McNulty P., Taegtmeyer H. (2002). Adaptation and maladaptation of the heart in diabetes: Part II: Potential mechanisms. Circulation.

[91] Churchill E.N., Murriel C.L., Chen C.H., Mochly-Rosen D., Szweda L.I. (2005). Reperfusion-induced translocation of δPKC to cardiac mitochondria prevents pyruvate dehydrogenase reactivation. Circ. Res..

[92] Ringvold H.C., Khalil R.A. (2017). Protein kinase C as regulator of vascular smooth muscle function and potential target in vascular disorders. Adv. Pharmacol..

[93] Chong C.R., Clarke K., Levelt E. (2017). Metabolic remodelling in diabetic cardiomyopathy. Cardiovasc. Res..

[94] Li Q., Park K., Li C., Rask-Madsen C., Mima A., Qi W., Mizutani K., Huang P., King G.L. (2013). Induction of vascular insulin resistance, endothelin-1 expression and acceleration of atherosclerosis by the overexpression of protein kinase C β isoform in the endothelium. Circ. Res..

[95] Roul D., Recchia F.A. (2015). Metabolic alterations induce oxidative stress in diabetic and failing hearts: Different pathways, same outcome. Antioxid. Redox Signal..

[96] Dai Ly L., Xu S., Choi S.K., Ha C.M., Thoudam T., Cha S.K., Wiederkehr A., Wollheim C.B., Lee I.K., Park K.S. (2017). Oxidative stress and calcium dysregulation by palmitate in type 2 diabetes. Exp. Mol. Med..

[97] Guertl B., Noehammer C., Hoefler G. (2000). Metabolic cardiomyopathies. Int. J. Exp. Pathol..

[98] O’Connell R.P., Musa H., San Martin Gomez M., Mahesh Avula U.M., Herron T.J., Kalifa J., Anumonwo J.M.B. (2015). Free fatty acid effects on the atrial myocardium: Membrane ionic currents are remodeled by the disruption of T-tubular architecture. PLoS ONE.

[99] Saha A.K., Vavvas D., Kurowski T.G., Apazidis A., Witters L.A., Shafrir E., Ruderman N.B. (1997). Malonyl-CoA regulation in skeletal muscle: Its link to cell citrate and the glucose-fatty acid cycle. Am. J. Physiol..

[100] Deblon N., Bourgoin L., Veyrat-Durebex C., Peyrou M., Vinciguerra M., Caillon A., Maeder C., Fournier M., Montet X., Rohner-Jeanrenaud F. (2012). Chronic mTOR inhibition by rapamycin induces muscle insulin resistance despite weight loss in rats. Br. J. Pharmacol..

[101] Fajemiroye J.O., da Cunha L.C., Saavedra-Rodríguez R., Rodrigues K.L., Naves L.M., Mourão A.A., da Silva E.F., Williams N.E.E., Rodrigues Martins J.L., Sousa R.B. (2018). Aging-Induced Biological Changes and Cardiovascular Diseases. Biomed. Res. Int..

[102] Balcıoğlu A.S., Müderrisoğlu H. (2015). Diabetes and cardiac autonomic neuropathy: Clinical manifestations, cardiovascular consequences, diagnosis and treatment. World J. Diabetes.

[103] Nascimben L., Ingwall J.S., Lorell B.H., Pinz I., Schultz V., Tornheim K., Tian R. (2004). Mechanisms for increased glycolysis in the hypertrophied rat heart. Hypertension.

[104] Sasaki H., Asanuma H., Fujita M., Takahama H., Wakeno M., Ito S., Ogai A., Asakura M., Kim J., Minamino T. (2009). Metformin prevents progression of heart failure in dogs: Role of AMP-activated protein kinase. Circulation.

[105] Bertrand L., Ginion A., Beauloye C., Hebert A.D., Guigas B., Hue L., Vanoverschelde J.L. (2006). AMPK activation restores the stimulation of glucose uptake in an in vitro model of insulin-resistant cardiomyocytes via the activation of protein kinase *B*. Am. J. Physiol. Heart Circ. Physiol..

[106] An D., Kewalramani G., Chan J.K.Y., Qi D., Ghosh S., Pulinilkunnil T., Abrahani A., Innis S.M., Rodrigues B. (2006). Metformin influences cardiomyocyte cell death by pathways that are dependent and independent of caspase-3. Diabetologia.

[107] Benes J., Kazdova L., Drahota Z., Houstek J., Medrikova D., Kopecky J., Kovarova N., Vrbacky M., Sedmera D., Strnad H. (2011). Effect of metformin therapy on cardiac function and survival in a volume-overload model of heart failure in rats. Clin. Sci..

[108] Chan A.Y., Soltys C.L., Young M.E., Proud C.G., Dyck J.R. (2004). Activation of AMP-activated protein kinase inhibits protein synthesis associated with hypertrophy in the cardiac myocyte. J. Biol. Chem..

[109] Chan A.Y., Dolinsky V.W., Soltys C.L., Viollet B., Baksh S., Light P.E., Dyck J.R. (2008). Resveratrol inhibits cardiac hypertrophy via AMP-activated protein kinase and Akt. J. Biol. Chem..

[110] Gal-Ben-Ari S., Kenney J.W., Ounalla-Saad H., Taha E., David O., Levitan D., Gildish I., Panja D., Pai B., Wibrand K. (2012). Consolidation and translation regulation. Learn. Mem..

[111] Wang X., Li W., Williams M., Terada N., Alessi D.R., Proud C.G. (2001). Regulation of elongation factor 2 kinase by p90^RSK1^ and p70 S6 kinase. EMBO J..

[112] Proud C.G. (1996). p70 S6 kinase: An enigma with variations. Trends Biochem. Sci..

[113] Gundewar S., Calvert J.W., Jha S., Toedt-Pingel I., Ji S.Y., Nunez D., Ramachandran A., Anaya-Cisneros M., Tian R., Lefer D.J. (2009). Activation of AMP-activated protein kinase by metformin improves left ventricular function and survival in heart failure. Circ. Res..

[114] Chang J.C., Kou S.J., Lin W.T., Liu C.S. (2010). Regulatory role of mitochondria in oxidative stress and atherosclerosis. World J. Cardiol..

[115] Harumi Tengan C., Silva Rodrigues G., Oliveira Godinho R. (2012). Nitric oxide in skeletal muscle: Role on mitochondrial biogenesis and function. Int. J. Mol. Sci..

[116] Vettor R., Valerio A., Ragni M., Trevellin E., Granzotto M., Olivieri M., Tedesco L., Ruocco C., Fossati A., Fabris R. (2014). Exercise training boosts eNOS-dependent mitochondrial bio-genesis in mouse heart: Role in adaptation of glucose metabolism. Am. J. Physiol. Endocrinol. Metab..

[117] Pechánová O., Varga Z.V., Cebová M., Giricz Z., Pacher P., Ferdinandy P. (2015). Cardiac NO signalling in the metabolic syndrome. Br. J. Pharmacol..

[118] Saito T., Hu F., Tayara L., Fahas L., Shennib H., Giaid A. (2002). Inhibition of NOS II prevents cardiac dysfunction in myocardial infarction and congestive heart failure. Am. J. Physiol. Heart Circ. Physiol..

[119] Yang B., Larson D.F., Watson R.R. (2004). Modulation of iNOS activity in age-related cardiac dysfunction. Life Sci..

[120] Förstermann U., Kleinert H. (1995). Nitric oxide synthase: Expression and expressional control of the three isoforms. Naunyn. Schmiedebergs. Arch. Pharmacol..

[121] Bułdak Ł., Łabuzek K., Bułdak R.J., Kozłowski M., Machnik G., Liber S., Suchy D., Duława-Bułdak A., Okopień B. (2014). Metformin affects macrophages’ phenotype and improves the activity of glutathione peroxidase, superoxide dismutase, catalase and decreases malondialdehyde concentration in a partially AMPK-independent manner in LPS-stimulated human monocytes/macrophages. Pharmacol. Rep..

[122] Tsujino M., Hirata Y., Imai T., Kanno K., Eguchi S., Ito H., Marumo F. (1994). Induction of nitric oxide synthase gene by interleukin-1 beta in cultured rat cardiocytes. Circulation.

[123] Davis B.J., Xie Z., Viollet B., Zou M.H. (2006). Activation of the AMP-activated kinase by antidiabetes drug metformin stimulates nitric oxide synthesis in vivo by promoting the association of heat shock protein 90 and endothelial nitric oxide synthase. Diabetes.

[124] Wang X.F., Zhang J.Y., Li L., Zhao X.Y., Tao H.L., Zhang L. (2011). Metformin improves cardiac function in rats via activation of AMP-activated protein kinase. Clin. Exp. Pharmacol. Physiol..

[125] Jyothirmayi G.N., Soni B.J., Masurekar M., Lyons M., Regan T.J. (1998). Effects of metformin on collagen glycation and diastolic dysfunction in diabetic myocardium. J. Cardiovasc. Pharmacol. Ther..

[126] Xiao H., Ma X., Feng W., Fu Y., Lu Z., Xu M., Shen Q., Zhu Y., Zhang Y. (2010). Metformin attenuates cardiac fibrosis by inhibiting the TGFβ1-Smad3 signalling pathway. Cardiovasc. Res..

[127] Han A., Lu Y., Zheng Q., Zhang J., Zhao Y., Zhao M., Cui X. (2018). Qiliqiangxin attenuates cardiac remodeling via inhibition of TGF-β1/Smad3 and NF-κB signaling pathways in a rat model of myocardial infarction. Cell. Physiol. Biochem..

[128] Ashraf J.M., Ahmad S., Rabbani G., Hasan Q., Jan A.T., Lee E.J., Khan R.H., Alam K., Choi I. (2015). 3-Deoxyglucosone: A potential glycating agent accountable for structural alteration in H3 histone protein through generation of different AGEs. PLoS ONE.

[129] Brings S., Fleming T., Freichel M., Muckenthaler M.U., Herzig S., Nawroth P.P. (2017). Dicarbonyls and advanced glycation end-products in the development of diabetic complications and targets for intervention. Int. J. Mol. Sci..

[130] Singh V.P., Bali A., Singh N., Singh Jagg A. (2014). Advanced glycation end products and diabetic complications. Korean J. Physiol. Pharmacol..

[131] Nowotny K., Jung T., Höhn A., Weber D., Grune T. (2015). Advanced glycation end products and oxidative stress in type 2 diabetes mellitus. Biomolecules.

[132] Li J., Schmidt A.M. (1997). Characterization and functional analysis of the promoter of RAGE, the receptor for advanced glycation end products. J. Biol. Chem..

[133] Dhingra R., Vasan R.S. (2012). Diabetes and the risk of heart failure. Heart Fail. Clin..

[134] Miki T., Yuda S., Kouzu H., Miura T. (2013). Diabetic cardiomyopathy: Pathophysiology and clinical features. Heart Fail. Rev..

[135] Beisswenger P.J., Howell S.K., Touchette A.D., Lal S., Szwergold B.S. (1999). Metformin reduces systemic methylglyoxal levels in type 2 diabetes. Diabetes.

[136] Scarpello J.H.B., Howlett H.C.S. (2008). Metformin therapy and clinical uses. Diab. Vasc. Dis. Res..

[137] Nobécourt E., Zeng J., Davies M.J., Brown B.E., Yadav S., Barter P.J., Rye K.A. (2008). Effects of cross-link breakers, glycation inhibitors and insulin sensitisers on HDL function and the non-enzymatic glycation of apolipoprotein A.-I. Diabetologia.

[138] Brown B.E., Mahroof F.M., Cook N.L., van Reyk D.M., Davies M.J. (2006). Hydrazine compounds inhibit glycation of low-density lipoproteins and prevent the in vitro formation of model foam cells from glycolaldehyde-modified low-density lipoproteins. Diabetologia.

[139] Machado A.P., Pinto R.S., Moysés Z.P., Nakandakare E.R., Quintão E.C., Passarelli M. (2006). Aminoguanidine and metformin prevent the reduced rate of HDL-mediated cell cholesterol efflux induced by formation of advanced glycation end products. Int. J. Biochem. Cell. Biol..

[140] Tanaka Y., Uchino H., Shimizu T., Yoshii H., Niwa M., Ohmura C., Mitsuhashi N., Onuma T., Kawamori R. (1999). Effect of metformin on advanced glycation endproduct formation and peripheral nerve function in streptozotocin-induced diabetic rats. Eur. J. Pharmacol..

[141] Skrha J., Prázný M., Hilgertová J., Kvasnicka J., Kalousová M., Zima T. (2007). Oxidative stress and endothelium influenced by metformin in type 2 diabetes mellitus. Eur. J. Clin. Pharmacol..

[142] Soraya H., Khorrami A., Garjani A., Maleki-Dizaji N., Garjani A. (2012). Acute treatment with metformin improves cardiac function following isoproterenol induced myocardial infarction in rats. Pharmacol. Rep..

[143] Khorrami A., Hammami M., Garjani M., Maleki-Dizaji N., Garjani A. (2014). Tacrolimus ameliorates functional disturbances and oxidative stress in isoproterenol-induced myocardial infarction. Daru.

[144] Sun S.J., Wu X.P., Song H.L., Li G.Q. (2015). Baicalin ameliorates isoproterenol-induced acute myocardial infarction through iNOS, inflammation, oxidative stress and P38MAPK pathway in rat. Int. J. Clin. Exp. Med..

[145] Eurich D.T., Majumdar S.R., McAlister F.A., Tsuyuki R.T., Johnson J.A. (2005). Improved clinical outcomes associated with metformin in patients with diabetes and heart failure. Diabetes Care.

[146] MacDonald M.R., Eurich D.T., Majumdar S.R., Lewsey J.D., Bhagra S., Jhund P.S., Petrie M.C., McMurray J.J.V., Petrie J.R., McAlister F.A. (2010). Treatment of type 2 diabetes and outcomes in patients with heart failure: A nested case–control study from the U.K. General Practice Research Database. Diabetes Care.

[147] Aguilar D., Chan W., Bozkurt B., Ramasubbu K., Deswal A. (2011). Metformin use and mortality in ambulatory patients with diabetes and heart failure. Circ. Heart Fail..

[148] McAlister F.A., Eurich D.T., Majumdar S.R., Johnson J.A. (2008). The risk of heart failure in patients with type 2 diabetes treated with oral agent monotherapy. Eur. J. Heart Fail..

[149] Shah D.D., Fonarow G.C., Horwich T.B. (2010). Metformin therapy and outcomes in patients with advanced systolic heart failure and diabetes. J. Card. Fail..

[150] Holstein A., Stumvoll M. (2005). Contraindications can damage your health—Is metformin a case in point?. Diabetologia.

[151] Eurich D.T., Weir D.L., Majumdar S.R., Tsuyuki R.T., Johnson J.A., Tjosvold L., Vanderloo S.E., McAlister F.A. (2013). Comparative safety and effectiveness of metformin in patients with diabetes mellitus and heart failure. Systematic review of observational studies involving 34 000 patients. Circ. Heart Fail..

[152] Gilbert R.E., Krum H. (2015). Heart failure in diabetes: Effects of antihyperglycaemic drug therapy. Lancet.

[153] Seferović P.M., Petrie M.C., Filippatos G.S., Anker S.D., Rosano G., Bauersachs J., Paulus W.J., Komajda M., Cosentino F., de Boer R.A. (2018). Type 2 diabetes mellitus and heart failure: A position statement from the Heart Failure Association of the European Society of Cardiology. Eur. J. Heart Fail..

